# Neutrophils predominate the immune signature of cerebral thrombi in COVID-19 stroke patients

**DOI:** 10.1186/s40478-022-01313-y

**Published:** 2022-02-01

**Authors:** Angela Genchi, Aurora Semerano, Ghil Schwarz, Beatrice Dell’Acqua, Giorgia Serena Gullotta, Michela Sampaolo, Enzo Boeri, Angelo Quattrini, Francesca Sanvito, Susanna Diamanti, Andrea Bergamaschi, Stefano Grassi, Paola Podini, Pietro Panni, Caterina Michelozzi, Franco Simionato, Francesco Scomazzoni, Paolo Remida, Luca Valvassori, Andrea Falini, Carlo Ferrarese, Patrik Michel, Guillaume Saliou, Steven Hajdu, Simone Beretta, Luisa Roveri, Massimo Filippi, Davide Strambo, Gianvito Martino, Marco Bacigaluppi

**Affiliations:** 1 Neuroimmunology Unit, Institute of Experimental Neurology, San Raffaele Hospital, Via Olgettina 60, 20132 Milan, Italy; 2 Department of Neurology, San Raffaele Hospital, Milan, Italy; 3 University Vita-Salute San Raffaele, Milan, Italy; 4 Department of Neurology and Stroke Unit, ASST Grande Ospedale Metropolitano Niguarda, Milan, Italy; 5 Department of Pathology, San Raffaele Hospital, Milan, Italy; 6 Neuropathology Unit, San Raffaele Hospital, Milan, Italy; 7 Department of Neuroradiology, San Raffaele Hospital, Milan, Italy; 8 Department of Microbiology and Virology, San Raffaele Hospital, Milan, Italy; 9 Department of Medicine and Surgery, San Gerardo Hospital and Milano–Bicocca University, Milan, Italy; 10 Department of Neuroradiology, San Gerardo Hospital, Monza, Italy; 11 Stroke Center, Neurology Service, Lausanne University Hospital and University of Lausanne, Lausanne, Switzerland; 12 Service of Diagnostic and Interventional Radiology, Interventional Neuroradiological Unit, Lausanne University Hospital and University of Lausanne, Lausanne, Switzerland

**Keywords:** SARS-CoV2, COVID-19, Thrombosis, Neutrophils, Ischemic stroke, Endovascular treatment

## Abstract

Coronavirus disease 2019 (COVID-19) is associated with an increased risk of thrombotic events. Ischemic stroke in COVID-19 patients entails high severity and mortality rates. Here we aimed to analyze cerebral thrombi of COVID-19 patients with large vessel occlusion (LVO) acute ischemic stroke to expose molecular evidence for SARS-CoV-2 in the thrombus and to unravel any peculiar immune-thrombotic features. We conducted a systematic pathological analysis of cerebral thrombi retrieved by endovascular thrombectomy in patients with LVO stroke infected with COVID-19 (*n* = 7 patients) and non-covid LVO controls (*n* = 23). In thrombi of COVID-19 patients, the SARS-CoV-2 docking receptor ACE2 was mainly expressed in monocytes/macrophages and showed higher expression levels compared to controls. Using polymerase chain reaction and sequencing, we detected SARS-CoV-2 Clade20A, in the thrombus of one COVID-19 patient. Comparing thrombus composition of COVID-19 and control patients, we noted no overt differences in terms of red blood cells, fibrin, neutrophil extracellular traps (NETs), von Willebrand Factor (vWF), platelets and complement complex C5b-9. However, thrombi of COVID-19 patients showed increased neutrophil density (MPO^+^ cells) and a three-fold higher Neutrophil-to-Lymphocyte Ratio (tNLR). In the ROC analysis both neutrophils and tNLR had a good discriminative ability to differentiate thrombi of COVID-19 patients from controls. In summary, cerebral thrombi of COVID-19 patients can harbor SARS-CoV2 and are characterized by an increased neutrophil number and tNLR and higher ACE2 expression. These findings suggest neutrophils as the possible culprit in COVID-19-related thrombosis.

**Graphical Abstract:**

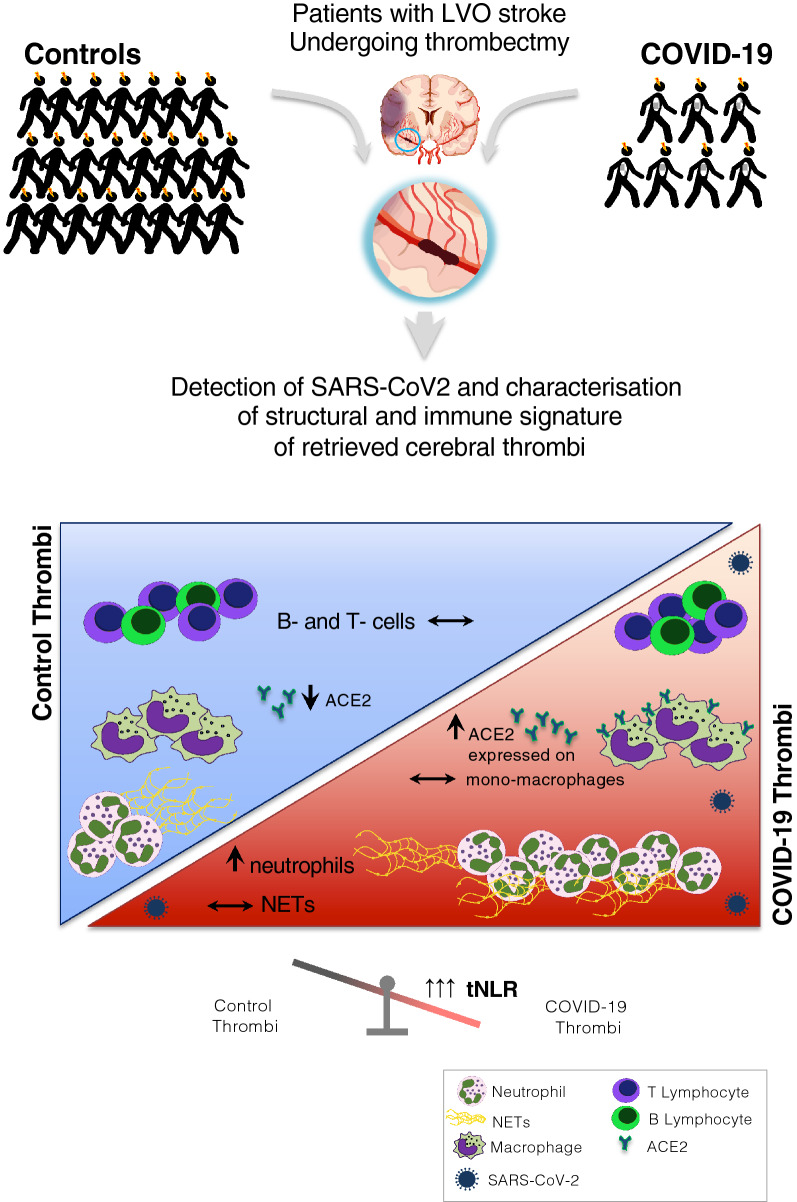

**Supplementary Information:**

The online version contains supplementary material available at 10.1186/s40478-022-01313-y.

## Introduction

Coronavirus disease 2019 (COVID-19) is primarily characterized by pulmonary involvement but neurological manifestations [10, 11, 14, 30, 39] and thrombotic complications [17] are also frequent. Previous observations and pathological descriptions reported both large vessel and microvascular thrombosis, suggesting peculiar prothrombotic pathophysiology of COVID-19 [9, 40]. Diverse pathophysiological mechanisms have been hypothesized to explain the prothrombotic state in COVID-19, namely direct viral invasion of vascular structures (such as endothelial cells) [37] or of blood cells, or as a consequence of the organ’s immune response. An excessive inflammatory response to SARS-CoV-2 associated with the development of coagulopathy are thought to be the most significant features of poor prognosis in COVID-19 patients [19, 36]. High D-dimer levels and coagulation abnormalities have been frequently reported in COVID-19 patients suggesting that the activation of the coagulation system may be involved as an effector pathway of the immune response to the virus. Angiotensin-converting enzyme 2 (ACE2)—the putative functional receptor for SARS-CoV-2 entry into host cells [15]—is mainly expressed in endothelial cells, macrophages and perivascular pericytes. Dysregulation of the angiotensin 2 (AngII)/angiotensin receptor type 1 (AT1R) pathway downstream of ACE2 could lead to cytokine release syndrome and severe endothelial dysfunction with consequent increased vascular permeability and uncontrolled inflammation, which could be implied in virus-specific thrombo-inflammatory mechanisms. Also, hypoxia, commonly observed in COVID-19 pneumonia, may induce a prothrombotic state by affecting coagulation and fibrinolysis pathways as well as endothelial and neutrophil functioning [29]. Furthermore, a hyper-inflammatory state with macrophage activation, hyperactivation of the myeloid compartment and cytokine storm has been observed in severe COVID-19 patients [33].

Ischemic stroke is not uncommon in patients with COVID-19, especially those with severe infection and pre-existing vascular risk factors [25]. A meta-analysis showed that acute cerebrovascular disease occurs in about 1.4% of the hospitalized COVID-19 population, with a prevalence of acute ischemic stroke over intracerebral hemorrhage [25]. Although the true relationship between COVID-19 and stroke incidence remains to be clarified, multicenter and meta-data suggest that ischemic stroke in COVID-19 patients is more severe with a worse functional outcome and higher mortality [21, 27].

The analysis of cerebral thrombi retrieved by endovascular procedures in patients with acute ischemic stroke from large vessel occlusion (LVO-AIS) has emerged as a tool for investigating the diverse pathophysiological mechanisms that contribute to thrombus formation [4, 34]. This may also apply to patients with LVO-AIS and concomitant COVID-19, whereby evaluating the composition of cerebral thrombi in terms of immune cells, endothelium and coagulation cascade components could provide new insights into the pathogenesis of SARS-CoV-2-related thrombosis and the association between ischemic stroke and SARS-CoV-2 infection.

In the present study, we analyzed cerebral thrombi retrieved after acute mechanical thrombectomy (MT) from AIS patients with COVID-19 and controls to provide a comprehensive description. We investigated, *i)* the expression of the SARS-CoV-2-docking receptor (ACE2) by cells within the thrombi, *ii)* the presence of SARS-CoV-2 within cerebral thrombi and *iii)* thrombus composition in terms of structural components (red blood cells, fibrin, von Willebrand Factor, platelets and complement) and the immune profile (neutrophils, macrophages, T and B-lymphocytes, and neutrophil extracellular traps [NETs] density).

## Methods

### Study population

The study was conducted on patients admitted to three comprehensive stroke centers in Italy and Switzerland (San Raffaele Hospital, Milan, Italy; San Gerardo Hospital, Monza, Italy; Lausanne University Hospital, Lausanne, Switzerland). From February 2020 to March 2021, we included prospective consecutive adult patients with COVID-19 and concomitant LVO-AIS treated with MT, and with a cerebral thrombus available for histological analysis. We defined a confirmed case of COVID-19 by the following criteria (± 15 days from the index event): 1.) clinical [8] and/or radiological [32, 38] features suggestive of COVID-19 infection (i.e. fever, dry cough, myalgias, dyspnea, hypo/anosmia, hypo/ageusia; peripheral ground glass lung opacities on chest x-rays and/or on chest CT scan); 2.) positive result for SARS-CoV-2 on real-time reverse- transcription polymerase chain reaction analysis of nasopharyngeal swab specimens and/or SARS-CoV-2 positive serology. Clinical and radiological features were respectively assessed as part of the standard clinical practice from treating physicians and expert radiologists.

In the primary analysis we selected a control group of COVID-19-negative (control) patients from consecutive LVO-AIS patients admitted to the San Raffaele Hospital between July 2016 and November 2019, treated by MT and with a thrombus available for analysis. Patients in the control group were matched to COVID-19 patients for stroke etiology, administration of intravenous thrombolysis (IVT) and anti-thrombotic drug at the index event.

In a secondary analysis, a distinct set of thrombi of LVO-AIS patients with recent pre-stroke infections (present at stroke symptom onset) and not related to SARS-CoV-2 was analysed. Recent pre-stroke infections were defined as suggestive symptoms (i.e., cough, dyspnea, pleuritic pain, urinary tract symptoms, etc.), history of fever within the previous 7 days of stroke symptom onset and/or determination of body temperature ≥ 37.5^◦^C at admission and white blood cell count ≥ 11, 000/mL or ≤ 4, 000/mL, pulmonary infiltrate on chest X-rays or cultures positive for a pathogen at admission [31].

The local Ethics Committees (Lausanne Hospital, San Raffaele Hospital, San Gerardo Hospital) approved the study and informed consent was collected. The research was conducted in compliance with international and community guidelines including the Convention on Human Rights and Biomedicine, the Council Recommendation of Europe on the protection of health data and the Helsinki declaration of the World Medical Association on principles for research involving human subjects.

### Clinical variables

For each included patient we collected: demographic data, vascular risk factors, prior anti-thrombotic therapy on admission, stroke severity (assessed by the National Institutes of Health Stroke Scale, NIHSS), administration of intravenous thrombolysis, early ischemic changes on non-contrast brain CT scan (assessed by the Alberta Stroke Program Early Computed Tomography Score, ASPECTS), vascular occlusion site, collateral grading score [35], details of the MT procedure (timings, number of maneuvers and type of device), degree of achieved reperfusion (by modified Treatment in Cerebral Infarction, mTICI scale), stroke etiology (according to the Trial of Org 10,172 in Acute Stroke Treatment (TOAST) [1] classification) and 3-month functional outcome (by modified Rankin Scale, mRS). Data from laboratory tests obtained up to 48 h from stroke symptoms onset were collected. Complete blood counts was assessed with an automated hemocytometer. We calculated the ratio between the absolute circulating neutrophil and lymphocyte count (blood neutrophil-to-lymphocyte ratio, NLR).

### Thrombi collection

Each participating center immediately fixed cerebral thrombi retrieved during the MT procedure in 10% formalin and stored them at + 4 °C until processing. Formalin-fixed specimens were then embedded in paraffin and cut into 5 μm serial sections. Thrombus analysis was performed centrally at the San Raffaele Scientific Institute, Milan, Italy, and included molecular biology, histology and electron microscopy analyses (details in the dedicated sections below). Thrombus analysis was performed in accordance with the guidelines of the institutional biosafety committee, with the use of proper personal protective equipment (PPE).

### Histology

Thrombus sections were stained with Hematoxylin and Eosin (H&E) and Lendrum (Martius Scarlet Blue, MSB). Red Blood cells (RBCs) and fibrin were quantified on MSB stained sections (yellow^+^ and fuchsia^+^ areas respectively). To quantify the other thrombus components of interest, we performed immunohistochemical staining using the following antibodies: anti-ACE2 (1:500, ThermoFisher, MA5-31,395), anti-CD34 (1:500, Invitrogen, MA1-10,202), anti-CD61 (1:100, Dako, M0753), anti-Von Willebrand Factor (1:1000, Abcam, ab6994), anti-MPO (1:500, Dako, A0398), anti-citH3 (1:200, Abcam, ab5103), anti-CD68 PG-M1 (1:75, Dako, M0978), anti-CD3 (clone 2GV6, Ventana, 790–4341), anti-CD20 (clone L26, Ventana,760–2531) and anti-C5b-9 (Abcam, ab55811). All stained slices were digitized using the Aperio® Microscope Digitizer (Leica Biosystems) at a magnification of 20x. For RBCs, fibrin, CD61, vWF, ACE2, CD34, citH3 and C5-C9b, the stained areas were quantified on at least two independent sections per thrombus using the Classification Algorithm on Orbit Image Analysis® (v3.64) and expressed as percentage of the total thrombus area. Immune cellular elements (ACE2^+^, CD34^+^, MPO^+^, CD68 PG-M1^+^, CD3^+^, and CD20^+^ cells) were counted using an object segmentation algorithm (Mumford-Shah segmentation algorithm) on Orbit Image Analysis® (v3.64) Software on at least two sections per thrombus and expressed as cells/mm^2^ [12].

### Electron microscopy

For transmission electron microscopy (TEM), we fixed small representative parts of the thrombus by immersion in 2.5% glutaraldehyde in 0.12 M sodium phosphate buffer overnight, then rinsing in the same buffer and immersing in 1% osmium tetroxide in 0.12 M sodium phosphate buffer for 2 h. We dehydrated tissues by a graded alcohol series and then infiltrated them with EPON Resin. Ultrathin sections of 60–70 nm were cut at using a microtome. We assessed sections with a Talos 120C (Fei) electron microscope.

### Molecular biology

To detect SARS-CoV-2 in fragments of formalin-fixed paraffin-embedded thrombi (*n* = 6), we extracted total RNA using the QIAamp DNA FFPE Tissue Kit (Qiagen) according to the manufacturer’s instructions. For one thrombus (*n* = 1) it was possible to search for SARS-CoV-2 RNA using a not paraffin-embedded thrombus fragment; in this case, we extracted total RNA with the RNeasy Lipid Tissue Mini kit (Qiagen) using an elution volume of 50 μL. RNA concentration and quality were measured. We tested RNA samples for presence of SARS- CoV-2 virus using the forward primer (5ʹCAAGTGGGGTAAGGCTAGACTTT-3ʹ) and reverse primer (5ʹ-ACTTAGGATAATCCCAACCCAT-3ʹ) recognizing a 344 bp sequence of the RNA-dependent RNA polymerase (RdRp) gene present in all severe acute respiratory syndrome (SARS)-related coronaviruses [7]. We performed reverse transcription and subsequent amplification using the SuperScript™ III One-Step RT-PCR System with Platinum™ Taq DNA Polymerase (ThermoFisher Scientific). We analyzed PCR products by electrophoresis on 1.5% agarose gels and confirmed specificity for SARS-CoV-2 in a sample by Sanger sequencing. For this, we purified the amplicon using HT ExoSAP-IT (Thermo Fischer Scientific) according to the manufacturer’s instructions and then performed sequencing using the BigDye Terminator kit v. 3.1 and cleaning with the BigDye XTerminator Purification Kit (Applied Biosystems Foster City, CA, USA). We analyzed purified products of the sequencing cycle on the ABI PRISM 3130xl Genetic Analyzer (Applied Biosystems) and generated nucleotide sequences with SeqScape®Software (ThermoFisher Scientific, Waltham, MA, USA).

### Statistical analysis

We described categorical variables by frequencies and percentages, and continuous variables using mean and standard deviation (SD) or median with interquartile range (IQR). We performed univariate comparison of clinical variables, laboratory results and thrombus parameters between COVID-19 and control groups using the Fisher’s exact test for categorical variables, Student’s t-test for normally distributed continuous variables and Mann–Whitney U test for non-normally distributed variables. Correlations were assessed using Spearman’s rank correlation coefficient.

To assess the performance of the different thrombus components to distinguish between COVID-19 and control patients, we built a receiver operating characteristic curve (ROC) with the thrombus component of interest as predicting variable and COVID-19 status as response variable. We assessed discrimination by calculating the area under the curve ROC (AUC-ROC).

We performed statistical analyses either with R statistical software (version3.3.2, R Core Team [2016], STATA 16) or with GraphPad Prism (version 8.0).

## Results

### Clinical, radiological and laboratory characteristics

During the study period, we included seven COVID-19 patients with LVO ischemic stroke treated by MT (mean age 70.9 years [± 12.4]; 42.9% females). The median delay between the beginning of COVID-19 symptoms and stroke onset was 5 days (IQR 3–10). The most frequent COVID-19 symptoms were fever (85.7%), cough (42.9%) and dyspnea (28.6%). All seven patients had lung opacities typical of COVID-19 on pulmonary imaging (chest X-ray or CT scan). We included a control group of 23 LVO stroke patients without SARS-CoV-2 infection. The baseline clinical and radiological features of the COVID-19 patients and controls are displayed in Table 1. COVID-19 patients had more severe strokes than controls (median baseline NIHSS: 24 [IQR 20–26] vs. 16 [IQR 9–22], respectively; p = 0.056). They had a similar rate of successful recanalization to control patients (mTICI ≥ 2b 71.4% vs. 82.6% respectively, p = 0.603), a similar time of symptom-to-groin-puncture (onset-to-groin puncture in minutes, 330 [255–495] vs 280 [193–675], respectively p = 0.556), but a greater number of device passes (first pass recanalization in 0% vs. 43.5%; need for ≥ 5 passes in 57.1% vs. 8.7% respectively, p = 0.012). COVID-19 patients had a higher rate of functional dependency at 3 months (mRS ≥ 3: 85.7% vs. 34.8% for controls; *p* = 0.018). No significant differences were found between the two groups in terms of blood leukocyte and blood neutrophil counts or the blood neutrophil-to-lymphocyte ratio (Table 1).

**Table 1:** General clinical, radiological and laboratory characteristics for the entire cohort and comparison between COVID-19 patients and controls

	Entire cohort *N* = 31 (%)	COVID-19 *N* = 7 (%)	Control group *N* = 23 (%)	*P* value
*Demographics*				
Age (mean ± SD)	73.8 ± 10.2	70.9 ± 12.4	74.7 ± 9.6	0.394
Female	16 (53.3)	3 (42.9)	13 (56.5)	0.675
*Medical history*				
Atrial fibrillation	9 (30.0)	3 (42.9)	6 (26.1)	0.640
Arterial hypertension	21 (70.0)	2 (28.6)	19 (82.6)	0.014^*^
Diabetes mellitus	4 (13.3)	0 (0)	4 (17.4)	0.548
Dyslipidemia	9 (30.0)	2 (28.6)	7 (30.4)	1.000
Current smoking	3 (10.0)	1 (14.3)	2 (8.7)	1.000
Previous AIS	7 (23.3)	2 (28.6)	5 (21.7)	1.000
*Previous antithrombotic medication*				
None	17 (56.7)	3 (42.9)	14 (60.9)	
Antiplatelet	4 (13.3)	1 (14.2)	3 (13.0)	0.832
Anticoagulant	9 (30.0)	3 (42.9)	6 (26.1)	
*Index ischemic event*				
Baseline NIHSS score (median [IQR])	18 (11 – 24)	24 (20 – 26)	16 (9 – 22)	0.056
Prestroke mRS > 2	3 (10.0)	1 (14.3)	2 (8.7)	1.000
ASPECTS (median [IQR])	9 (7 – 10)	9 (7 – 10)	9 (7 – 10)	0.937
*Occlusion site*				
Proximal anterior circulation^§^	26 (86.6)	5 (71.4)	21 (91.3)	
Tandem occlusion	2 (6.7)	2 (28.6)	0 (0.0)	0.095
Posterior circulation	2 (6.7)	0 (0.0)	2 (8.7)	
*Collateral status*^*$*^				
Absent	3 (10.0)	2 (28.6)	1 (4.4)	
Mild	3 (10.0)	0 (0.0)	3 (13.0)	
Intermediate	14 (46.7)	4 (57.1)	10 (43.5)	0.141
Good	7 (23.3)	0 (0.0)	7 (30.4)	
Not applicable	3 (10.0)	1 (14.3)	2 (8.7)	
*TOAST classification*				
ESUS	10 (33.3)	2 (28.6)	8 (34.8)	
LAA	9 (30.0)	2 (28.6)	7 (30.4)	1.000
CE	11 (36.7)	3 (42.8)	8 (34.8)	
*Laboratory findings*				
Leukocytes (× 10^3^/μL) (Mean ± SD)	8.7 ± 3.0	8.9 ± 4.4	8.6 ± 2.6	0.794
Neutrophils (× 10^3^/μL) (Mean ± SD)	7.4 ± 2.9	8.1 ± 4.0	7.2 ± 2.5	0.521
Eosinophils (× 10^3^/μL) (Median [IQR])	0.0 [0.0–0.1]	0.0 [0.0–0.0]	0.05 [0.0–0.1]	0.188
Lymphocytes (× 10^3^/μL) (Median [IQR])	1.3 [1.0–1.7]	0.9 [0.6–1.7]	1.3 [1.2–1.8]	0.078
NLR (ratio) (Median [IQR])	5.8 [3.8–8]	8.5 [2.3–23.5]	5.3 [4.1–6.2]	0.176
Monocytes (× 10^3^/μL) (Median [IQR])	0.8 [0.7–1.0]	0.8 [0.7–1.0]	0.8 [0.7–1.0]	0.877
Platelet count (× 10^3^/μL) (Median [IQR])	225 [172–260]	229 [199–346]	214 [169–255]	0.218
INR (Median [IQR])	1.1 [1.0–1.5]	1.1 [1.0–1.2]	1.1 [1.1–2.0]	0.218
D-Dimer (µg/mL) (Median [IQR])	1.3 [0.8–2.5]	1.2 [0.9–18.4]	1.3 [0.8–2.5]	0.752
Fibrinogen (mg/mL) (Mean ± SD)	355 ± 155	272 ± 285	375 ± 108	0.187
CRP (mg/L) (Median [IQR])	3.2 [1.2–18.2]	16.5 [1.2–56.1]	3.2 [0.9–9.1]	0.316
*Acute treatment*				
Intravenous Alteplase	9 (30.0)	2 (28.6)	7 (30.4)	1.000
Onset to groin time (median [IQR])	280 (235 – 570)	330 (255–495)	280 (193–675)	0.556
*Number of passages*				
1	10 (33.3)	0 (0.0)	10 (43.5)	
2–4	14 (46.7)	3 (42.9)	11 (47.8)	0.012^*^
≥ 5	6 (20.0)	4 (57.1)	2 (8.7)	
*Devices*				
Aspiration	13 (43.3)	2 (28.6)	11 (47.8)	
Stent retriever	3 (10.0)	2 (28.6)	1 (4.4)	0.233
Combined technique	14 (46.7)	3 (42.8)	11 (47.8)	
mTICI ≥ 2b	24 (80.0)	5 (71.4)	19 (82.6)	0.603
*Outcome*				
90-day mRS > 2	14 (46.7)	6 (85.7)	8 (34.8)	0.018^*^

Abbreviations. SD, standard deviation; AIS, acute ischemic stroke; ESUS, embolic stroke of undetermined source; LAA, large‐artery atherosclerosis; CE, cardioembolism; *Abbreviations. SD, standard deviation; NLR, neutrophil to lymphocyte ratio; INR, international normalized ratio; CRP, C-reactive protein.*

^§^Proximal anterior circulation includes: intracranial ICA and M1 segment of middle cerebral artery

^$^Collaterals were assessed with Tan collateral score

*Statistically significant

### ACE2 expression is increased in cerebral thrombi of COVID-19 patients

**Fig. 1:**
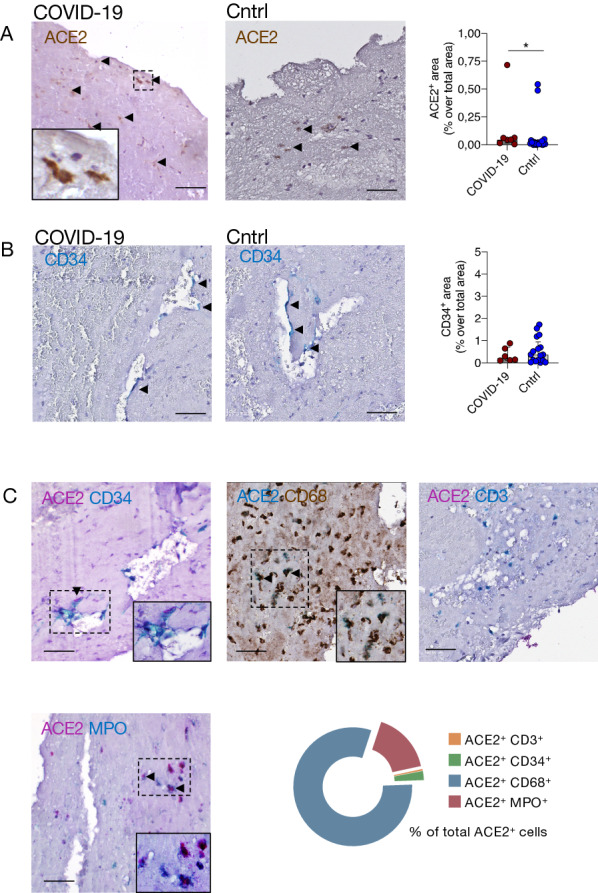
Evaluation of ACE2 protein and endothelial cells in cerebral thrombi. **A** ACE2 expression in thrombi of COVID-19 patients and controls. Representative images of a thrombus of a COVID-19 and a control patient with ACE2 immunohistochemical staining (staining in brown, the inset displays a magnification of the dashed box area and arrowheads highlight some positive cells). Histogram showing the ACE2^+^ area as percentage of the total thrombus area, *p = 0.04, Mann–Whitney. **B.** Endothelial cells (CD34^+^ area) in cerebral thrombi of COVID-19 patients and controls. Representative images of a thrombus of a COVID-19 and control patient with CD34 immunohistochemical staining (in blue, arrowheads highlight CD34^+^ cells). Histogram of the CD34^+^ area as percentage of the total thrombus area; *p* = 0.86, Mann–Whitney. **C.** Characterization of ACE2-expressing cells. Representative images of double-immunohistochemistry with ACE2 (in either purple or blue) and either CD34 (in blue), CD68 (in brown), CD3 (in blue), or MPO (in blue). The inset displays a magnification of the dashed box area and arrowheads highlight double positive cells. A donut graph showing the percentage of cells expressing ACE2 out of the total of ACE2^+^ cells. The graphs represent the median and IQR, each dot in the scatter plot represents the thrombus of one patient (*n* = 7 COVID-19 patients and *n* = 23 controls); scale bar A- C, 50 µm

Thrombi showed few ACE2 positive (ACE2^+^) cells, although a higher ACE2^+^ area in thrombi of COVID-19 patients was apparent compared to controls (respectively, median ACE2^+^ area as % over total thrombus area = 0.05%, [IQR 0.02–0.06] vs. 0.01%, [IQR 0.01–0.03], p = 0.04) (Fig. 1A). As endothelial cells are one of the cell types known to express ACE2 and recent reports described SARS-CoV-2 in endothelial cells [37], we assessed endothelial cell numbers in the cerebral thrombi. We observed a scant quantity of non-organized endothelial cells in all thrombi, without differences between COVID-19 and control groups (respectively, median CD34^+^ area over total thrombus area = 0.23%, [IQR 0.12–0.71] vs. 0.37%, [IQR 0.10–0.95], *p* = 0.86) (Fig. 1B). Subsequently, to characterize the cellular populations expressing ACE2 within the thrombi, we performed double-immunostaining with antibodies against ACE2 and markers of T lymphocytes, endothelial cells, monocytes/macrophages or neutrophils (respectively, CD3^+^, CD34^+^, CD68^+^ or MPO^+^). We found that most ACE2^+^ cells were monocytes/macrophages (80.6% of ACE2^+^CD68^+^ cells out of all ACE2^+^ cells) (Fig. 1C). In summary, we found that the thrombi of COVID-19 patients showed a higher number of ACE2^+^ cells compared to controls. In both groups, the ACE2 positive cells were predominantly monocytes/macrophages.

### SARS-CoV-2 within the retrieved thrombi of COVID-19 stroke patients

**Fig. 2:**
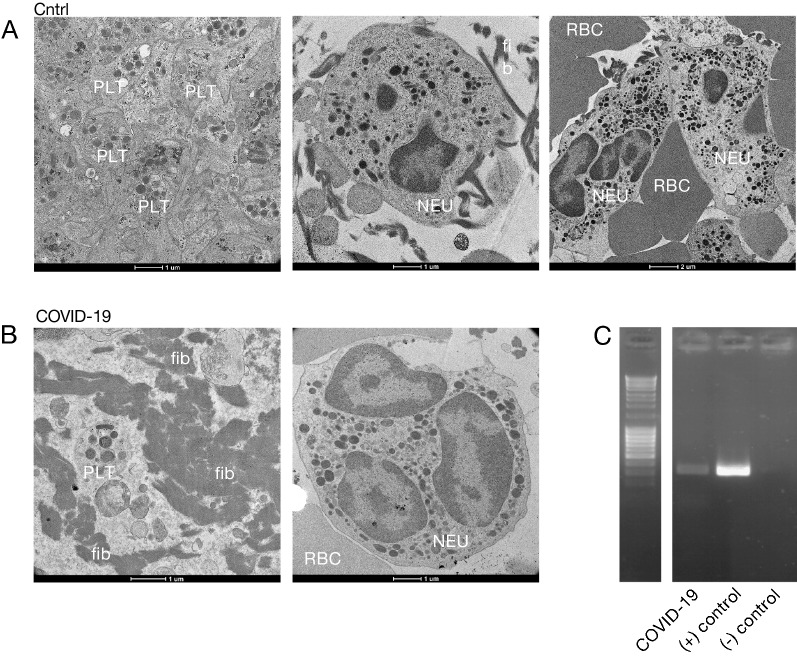
SARS-CoV-2 detection within the retrieved thrombus of COVID-19 stroke patients. **A.** Transmission Electron Microscopy images of a cerebral thrombus of a control and **B.** of COVID-19 stroke patient. We did not detect evidence of SARS-CoV2 viral particles within the analyzed thrombi. The white labels in the images indicate, platelets (PLT), neutrophils (NEU), red blood cells (RBC, fibrin (fib); **C**. Agarose gel showing the PCR amplification of SARS-CoV2 in a thrombus of a COVID-19 stroke patient. A negative and positive control for the PCR are shown

Next, we investigated molecular evidence for SARS-CoV-2 in cerebral thrombi of COVID-19 patients using a multimodal approach. In transmission electron microscopy (TEM), there was no evidence of obvious particles resembling SARS-CoV-2 in the analyzed thrombi (*n* = 7 thrombi of COVID-19 patients and *n* = 4 thrombi of control group) (Fig. 2a–b). However, we were able to successfully identify SARS-CoV-2 RNA by polymerase-chain-reaction (PCR) in one thrombus of a COVID-19 patient, while in the other six thrombi of COVID-19 patients and thrombi of controls, no SARS-CoV-2 RNA could be detected by PCR (Fig. 2C).

Importantly, we further confirmed the viral PCR detection by sequencing, which revealed presence of the SARS-CoV-2 clade 20A, identified by NextClade v 1.6.0 (clades.nextstrain.org) (data not shown). Overall, we found that we could detect SARS-CoV-2, although rarely, within retrieved thrombi of COVID-19 patients.

### Structural analysis and immune phenotyping of cerebral thrombi of COVID-19 patients and controls

**Fig. 3:**
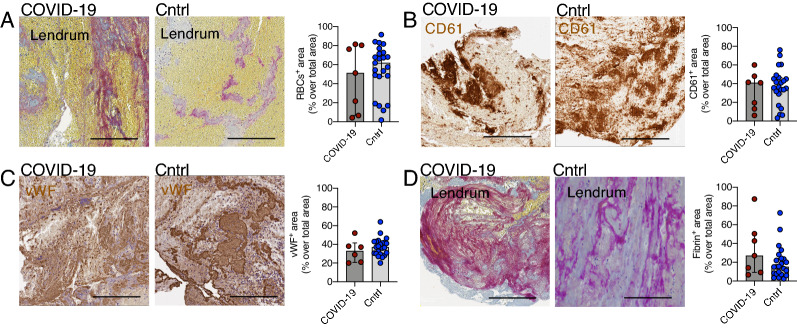
Analysis of major components of thrombi of COVID-19 and control stroke patients. **A**. Representative Lendrum (MSB) staining highlighting in yellow the red cell blood component in a COVID-19 and a control thrombus and quantification; *p* = 0.666, Mann Whitney. **B**. Representative images of platelets with CD61 immunohistochemical staining (in brown, and quantification; *p* = 0.69, Mann Whitney). **C**. Representative images of von Willebrand Factor (vWF in brown) immunohistochemistry and quantification; *p* = 0.348, Mann Whitney. **D**. Representative images of a COVID-19 and control thrombus with Lendrum (MSB) staining for fibrin identification (in pink) and quantification; p = 0.266, Mann Whitney. The graphs represent the median and IQR, each dot in the scatter plot represents the thrombus of one patient (*n* = 7 COVID-19 patients and *n* = 23 controls); scale bar in **A–F,** 100 μm

**Fig. 4:**
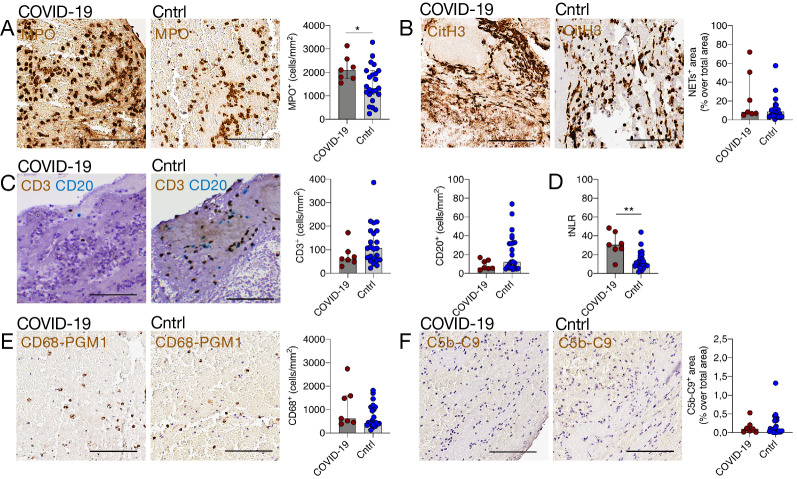
Immune signature of thrombi of COVID-19 and control stroke patients. **A to F.** Characterization of the thrombus immune cell signature of thrombi retrieved from COVID-19 and control stroke patients. **A.** Neutrophil number (MPO^+^ cells) and representative images of a thrombus of a COVID-19 and control patient with MPO immunohistochemical staining (in brown; *p* = 0.04, Mann Whitney). **B.** NET content (CitH3^+^ area) and representative images of thrombi with citH3 immunohistochemical staining (in brown; % of citH3^+^ area out of total thrombus area in COVID-19 patients and controls respectively, median [IQR]; *p* = 0.19, Mann Whitney). **C**. T and B cells (CD3+ and CD20+ cells) number and representative images of thrombi with CD3 (in brown) and CD20 (in blue) immunohistochemical staining of COVID-19 and control stroke patients; *p* = 0.11, median [IQR], Mann Whitney). **D**. Histogram representing the thrombus neutrophil-to-lymphocyte ratio (tNLR, in COVID-19 and control patients; *p* ≤ 0.01, Mann Whitney). **E**. Macrophages (CD68 PGM1^+^ cells) and representative images of thrombi with CD68-PGM1 immunohistochemical staining (in brown; *p* = 0.12, Mann Whitney). **F**. Quantification of complement (C5b-C9^+^ area) and representative images of thrombi with C5b-C9 immunohistochemical staining (in brown). Scale bar in**A–F**, 100 μm. In the scatter plots each dot corresponds to the thrombus of one patient (*n* = 7 COVID-19 and *n* = 23 control patients)

**Fig. 5:**
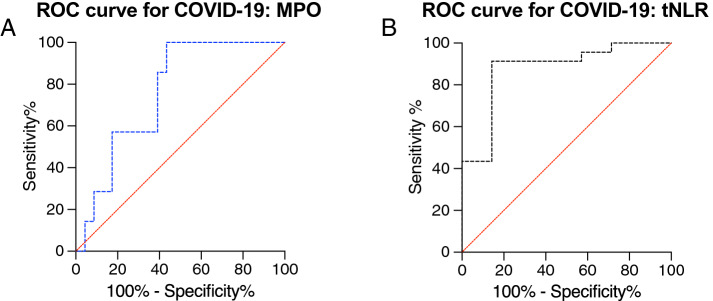
Receiver operating characteristic curves for COVID-19. **A**, **B** Receiver operating characteristic curves for COVID-19. Area under the curve (AUC) for COVID-19: AUC 0.758, confidence interval (CI) 0.583 to 0.933 for thrombus neutrophils (MPO+ cells) (**A**) and AUC 0.876, CI 0.719 to 1.000 for the thrombus neutrophil-to-lymphocyte ratio (**B**)

The composition of thrombi concerning red blood cells, platelets, von Willebrand Factor (vWF) and fibrin did not differ significantly between the two groups (Fig. 3A–D). The number of neutrophils (MPO^+^ cells) was significantly higher in thrombi of COVID-19 patients than controls (respectively, median MPO^+^ cells/mm^2^ = 2110, [IQR 1754–2580] vs. 1333, [IQR 1060–2082], p = 0.04) (Fig. 4A). On the contrary, the content of NETs was not significantly different between the two groups (in COVID-19, median citH3^+^ area was 8.66% of total area, [IQR 3.33–12.77] vs. 8.35%, [IQR 6.55–50.7] in controls, p = 0.19) (Fig. 4B). We observed no significant difference between T- and B- lymphocytes in thrombi of COVID-19 patients and controls (respectively, median CD3^+^ cells/mm^2^ = 61.6, [IQR 48.1–91.6] vs. 105.6, [IQR 57.4–179.9], p = 0.16, and median CD20^+^, cells/mm^2^ = 6.3, [IQR 5.2–14.3] vs. 12.4, [IQR 6.4–32.6], p = 0.11) (Fig. 4C). The neutrophil-to-lymphocyte ratio in the thrombus (tNLR, calculated as ratio of MPO^+^ cell/mm^2^ count over the sum of CD3^+^ and CD20^+^ cells/mm^2^) was significantly different between the groups: thrombi of COVID-19 patients had a three-fold higher tNLR than control patients (respectively, median tNLR 30.3, [IQR 25.9–44.4] vs. 10.7, [IQR 8.1–19.0], *p* ≤ 0.01), (Fig. 4D). The number of monocyte/macrophages did not differ between groups (in COVID-19 compared to the controls respectively, median CD68^+^ cells/mm^2^ = 623.4 [IQR 455.9–1588.0] vs. 438.8 [IQR 345.2–1107.0], p = 0.12). Thrombi of control and COVID-19 stroke patients were almost entirely negative for complement complex C5b-9, without a significant difference between groups. In the ROC analysis, neutrophils and tNLR had a good discriminative ability to differentiate thrombi of COVID-19 patients from controls with an AUC of 0.758, 95% CI [0.583- 0.933] and 0.876, 95% CI [0.719- 1.000], respectively. (Fig. 5A, B).

We did not find a correlation between blood and thrombus neutrophil numbers (r = 0.227; p = 0.254 Spearman’s coefficient) and there was no correlation between the NLR in blood and the tNLR (r = 0.183; p = 0.381 Spearman’s coefficient).

A secondary analysis comparing thrombi of COVID-19 patients with thrombi deriving from patients with non- SARS-CoV2 pre-existing infections at stroke onset, confirmed the increased neutrophil density and the higher tNLR in the COVID-19 group. We found no other significant differences regarding thrombus immune phenotype and composition between thrombi of patients with COVID-19 and with pre-existing infections (Additional file 1: Fig. 1 and Additional file 2: Table 1).

Altogether, thrombi of COVID-19 patients differ from those of controls in terms of an increased neutrophil content, particularly evident after calculating the thrombus neutrophil-to-lymphocyte ratio.

## Discussion

In this prospective multicenter study, cerebral thrombi of COVID-19 stroke patients featured an increased content of neutrophils and a higher neutrophil-to-lymphocyte ratio. We could detect SARS-CoV-2 directly in one thrombus of a COVID-19 patient, and ACE2 levels were higher in cerebral thrombi of COVID-19 patients compared to controls.

Our findings suggest that the endothelial cells within our cohort of cerebral thrombi have a limited significance as a direct target for SARS-CoV-2. Previous studies described local endotheliopathy as an important element in COVID-19-associated coagulopathy [13], however in our analyses we could not study the arterial wall where we extracted the thrombus from. Still, we observed that the increased expression of ACE2 in COVID-19 thrombi was mainly driven by a subset of CD68^+^ monocyte/macrophages within the thrombus. Recent reports have stated that inflammatory signals can trigger ACE2 expression, such as type I interferon [41]. Therefore, CD68^+^ monocytes/macrophages present in the thrombus might potentially represent a target for SARS-CoV-2 and a trigger for immune-induced thrombosis. Of note, despite a recent report showing ACE2 expression in a rare CD4^+^ T-cell subset, [18] in our study we almost never found lymphocytes expressing ACE2.

In this work, we report for the first-time proof that SARS-CoV-2 can be detected in the cerebral thrombotic material. Our finding was also confirmed by RNA sequencing, ruling out the possibility of a false-positive result. The limited detection rate of SARS-CoV-2 in the thrombi of COVID-19 patients may be due to technical issues, such as the formalin fixation (required in our institution for safety reasons), paraffin embedding and the scarcity of the virus in thrombi, possibly hampering the detection of SARS-CoV-2 in many cases. In addition, pathophysiological reasons may also play a role as COVID-19 may cause or trigger the stroke in some patients, but be an incidental concomitant comorbidity in others. A previous single-case report analyzing the cerebral thrombus of a COVID-19 patient could not detect the virus [5] in the thrombus nor in the endothelial cells of coronary heart vessels [28]. In another study on coronary thrombi however, thrombus viral load was found to be a possible determinant of the thrombus dimension independently of risk factors, and of poorer myocardial blush grade [20].

The composition of a thrombus has been described to be influenced by the underlying etiology of the stroke, the site of thrombus origin and the age of the thrombus [4]. While COVID-19 may trigger alterations in the coagulation cascade, in systemic inflammation and in endotheliopathy [2] [13, 22–24, 26], we did not find any significant difference in the content of red blood cells, platelets, fibrin and von Willebrand factor when comparing thrombi of the COVID-19 patients and controls. Contrary to a recent post-mortem study on COVID-19 patients that found a significant increase in fibrin and terminal complement C5b-C9 in heart microthrombi [28], we observed almost complete absence of C5b-C9, similar to the level the authors saw in larger coronary artery thrombus aspirates from COVID-19 STEMI cases [28].

On the other hand, analyzing the immune signature of the thrombus of COVID-19 stroke patients we found that thrombi of COVID-19 patients contain an increased density of neutrophils and a reduced level of lymphocytes compared to non-COVID-19 stroke patients. This difference becomes particularly remarkable on calculating the thrombus neutrophil-to-lymphocyte ratio. Indeed, in the ROC analysis, we found that the tNLR was the best predictor for discriminating the thrombi of the two groups of patients. We also confirmed the higher neutrophil density and tNLR of COVID-19 LVO patient thrombi compared to thrombi of stroke patients with pre-existing non-SARS-CoV-2 infections. Thrombus neutrophils and tNLR were not correlated with blood neutrophils or NLR, respectively; suggesting the thrombus is a site of active neutrophil recruitment and not a mere reflection of the blood cell content [12]. Similarly, a recent study on myocardial thrombi found increased markers of neutrophil activation in patients with COVID-19, including neutrophil-platelet aggregates and neutrophil-rich clusters in the macrothrombi [16]. Beyond a quantitative change in neutrophils, reports also describe a deranged phenotype and functionality in COVID-19 [6, 33]: the neutrophil activation signature shows prominent features of immature neutrophils in severe COVID-19 cases, representing a clear indication of emergency myelopoiesis [3]. Transcriptional and functional analyses of the neutrophil compartment in the blood of COVID-19 patients have shown an increased capacity for NET formation and enhanced cytokine production and calprotectin release [33]. However, in our study we did not find a significant change in NET density between thrombi of COVID-19 patients and controls, despite the increased neutrophil content. The limited number of thrombi of COVID-19 patients as well as the recently described heterogeneity of NETs in thrombi of diverse etiology [12] might have reduced the possibility of finding differences in NET density between our patient groups.

Our study has some limitations including the small sample size of COVID-19 thrombi analyzed, the limited immunophenotyping of the immune cell subpopulations and the difficulty of dissecting possible thrombus composition peculiarities from SARS-CoV2 compared to other viral infections despite the control group. The strength of this study is it brings the first thorough investigation of the microbiological, structural and inflammatory features of cerebral thrombi retrieved from patients with COVID-19 and large vessel occlusion stroke.

In conclusion, cerebral thrombi of COVID-19 patients can carry the SARS-CoV2 and have an increased neutrophil number, tNLR and ACE2 expression. These findings suggest that neutrophils are the possible culprit in COVID-19-related thrombosis.

## Supplementary Information

**Additional file 1: Figure S1**. Structural and immune characterization of thrombi of stroke patients with COVID-19 or pre-existing infections. (**A**) Characterization of the structural components. RBCs+ area median [IQR] in thrombi of COVID-19: 62.9 [IQR 25.9-73.8] and pre-infections stroke patients: 42.6 [IQR 30.4-58.5]; p=0.73; CD61+ area in thrombi of COVID-19: 41 [IQR 13-48] and pre-infections stroke patients: 25 [IQR 15.1-43.9]; p=0.68; vWF+ area in thrombi of COVID-19: 33 [IQR 20.8-41.5] and pre-inf: 43 [IQR 35.3-50]; p=0.19; Fibrin+ area in COVID-19: 27.1 [IQR 9.2-50.4] and pre-infections stroke patients: 8.8 [IQR 2.6-33.4]; p=0.15, Mann Whitney. (**B**) Characterization of the immune cell signature. Neutrophil number, MPO+ cells/mm2, median [IQR], in thrombi of COVID-19: 2110 [IQR 1754-2580] and pre-infections stroke patients: 985.1 [IQR 690.7-1573] p=0.008; NET content, CitH3+ area in thrombi of COVID-19: 8.4 [IQR 6.6-50.7] and pre-infections stroke patients: 12.2 [IQR 4.1-32.2] p=0.69; T cells number, CD3+ cells/mm2 in thrombi of COVID-19: 61.6 [IQR 48.1-91.6] and pre-infections stroke patients: 117.4 [IQR 31.7-144.9] p=0.92; B cells number, CD20+ cells/mm2 in thrombi of COVID-19: 6.3 [IQR 5.2-14.3] and pre-infections stroke patients: 20.8 [IQR 6.6-32.6] p=0.17, Mann Whitney. (**C**) Neutrophil to lymphocyte ratio (tNLR) in thrombi of COVID-19: 30.3 [IQR 25.9-44.4] and pre-infections stroke patients: 8.2 [IQR 5.9-19.1] p=0.02; macrophages number, CD68 PGM1+ cells/mm2 in thrombi of COVID-19: 623.4 [IQR 455.9-1588] and pre-infections stroke patients: 580 [IQR 206.7-1639] p=0.54; complement C5b-C9+ area in thrombi of COVID-19: 0.1028 [IQR 0.013- 0.206] and pre-infections stroke patients: 0.037 [IQR 0.004- 0.762] p=0.918, Mann Whitney. C. Quantification of ACE2 (median [IQR]) ACE2+ area in thrombi of COVID-19: 0.05 [IQR 0.01-0.06] and pre-infections stroke patients: 0.5 [IQR 0.02-1.7] p=0.34 and endothelial cells, CD34+ area in thrombi of COVID-19: 0.23 [IQR 0.12-0.71] and pre-infections stroke patients: 0.07 [IQR 0.05-0.24] p=0.06, Mann Whitney. The graphs represent the median and IQR, each dot in the scatter plot represents the thrombus of one patient, n=7 and n=8-10 for thrombi of COVID-19 and pre-infection stroke patients respectively.

**Additional file 2: Table S1**: General clinical, radiological and laboratory characteristics and comparison between COVID-19 patients and pre-infection LVO stroke patients.

## Data Availability

The data that support the findings of this study are available, upon reasonable request.
