# Supplementary figures and images for: Neutrophils predominate the immune signature of cerebral thrombi in COVID-19 stroke patients
Source: Acta Neuropathol Commun. 2022 Feb 1;10:14. doi: 10.1186/s40478-022-01313-y (PMC8805426; doi:10.1186/s40478-022-01313-y)

Figure S1

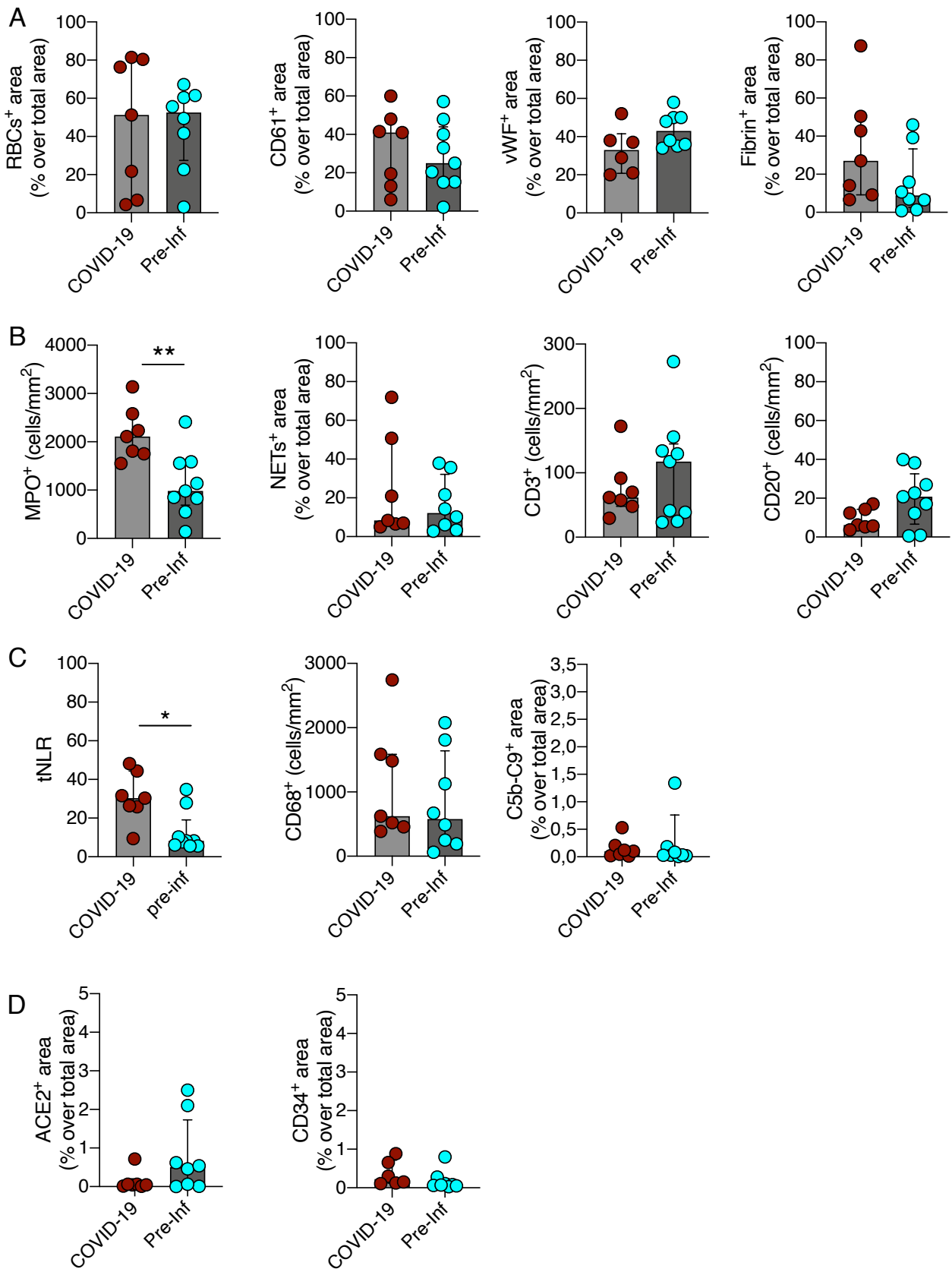

Supplement: Supplementary file 1 — Additional file 1: Figure S1. Structural and immune characterization of thrombi of stroke patients with COVID-19 or pre-existing infections. (A) Characterization of the structural components. RBCs+ area median [IQR] in thrombi of COVID-19: 62.9 [IQR 25.9-73.8] and pre-infections stroke patients: 42.6 [IQR 30.4-58.5]; p=0.73; CD61+ area in thrombi of COVID-19: 41 [IQR 13-48] and pre-infections stroke patients: 25 [IQR 15.1-43.9]; p=0.68; vWF+ area in thrombi of COVID-19: 33 [IQR 20.8-41.5] and pre-inf: 43 [IQR 35.3-50]; p=0.19; Fibrin+ area in COVID-19: 27.1 [IQR 9.2-50.4] and pre-infections stroke patients: 8.8 [IQR 2.6-33.4]; p=0.15, Mann Whitney. (B) Characterization of the immune cell signature. Neutrophil number, MPO+ cells/mm2, median [IQR], in thrombi of COVID-19: 2110 [IQR 1754-2580] and pre-infections stroke patients: 985.1 [IQR 690.7-1573] p=0.008; NET content, CitH3+ area in thrombi of COVID-19: 8.4 [IQR 6.6-50.7] and pre-infections stroke patients: 12.2 [IQR 4.1-32.2] p=0.69; T cells number, CD3+ cells/mm2 in thrombi of COVID-19: 61.6 [IQR 48.1-91.6] and pre-infections stroke patients: 117.4 [IQR 31.7-144.9] p=0.92; B cells number, CD20+ cells/mm2 in thrombi of COVID-19: 6.3 [IQR 5.2-14.3] and pre-infections stroke patients: 20.8 [IQR 6.6-32.6] p=0.17, Mann Whitney. (C) Neutrophil to lymphocyte ratio (tNLR) in thrombi of COVID-19: 30.3 [IQR 25.9-44.4] and pre-infections stroke patients: 8.2 [IQR 5.9-19.1] p=0.02; macrophages number, CD68 PGM1+ cells/mm2 in thrombi of COVID-19: 623.4 [IQR 455.9-1588] and pre-infections stroke patients: 580 [IQR 206.7-1639] p=0.54; complement C5b-C9+ area in thrombi of COVID-19: 0.1028 [IQR 0.013- 0.206] and pre-infections stroke patients: 0.037 [IQR 0.004- 0.762] p=0.918, Mann Whitney. C. Quantification of ACE2 (median [IQR]) ACE2+ area in thrombi of COVID-19: 0.05 [IQR 0.01-0.06] and pre-infections stroke patients: 0.5 [IQR 0.02-1.7] p=0.34 and endothelial cells, CD34+ area in thrombi of COVID-19: 0.23 [IQR 0.12-0.71] and [file 40478_2022_1313_MOESM1_ESM.pdf]
