# Supplementary material for: Neutrophils predominate the immune signature of cerebral thrombi in COVID-19 stroke patients
Source: Acta Neuropathol Commun. 2022 Feb 1;10:14. doi: 10.1186/s40478-022-01313-y (PMC8805426; doi:10.1186/s40478-022-01313-y)
Supplement: Supplementary file 2 — Additional file 2: Table S1: General clinical, radiological and laboratory characteristics and comparison between COVID-19 patients and pre-infection LVO stroke patients. [file 40478_2022_1313_MOESM2_ESM.docx]

**Table 1. General clinical, radiological and laboratory characteristics and comparison between COVID-19 and pre-infection stroke patients.**

|  | **COVID-19**  **N = 7 (%)** | **Pre-infections**  **N=10 (%)** | **P value** |
| --- | --- | --- | --- |
| **Demographics** |  |  |  |
| **Age** (mean ± SD) | 70.9 ± 12.4 | 74.3 ±11.9 | 0.715 |
| **Female** | 3 (42.9) | 7 (70) | 0.350 |
| **Medical history** |  |  |  |
| **Atrial fibrillation** | 3 (42.9) | 2(20) | 0.593 |
| **Arterial hypertension** | 2 (28.6) | 9 (90) | **0.035** |
| **Diabetes mellitus** | 0 (0) | 2 (20) | 0.485 |
| **Dyslipidemia** | 2 (28.6) | 3 (30) | 1.000 |
| **Current smoking** | 1 (14.3) | 1(10) | 1.000 |
| **Previous AIS** | 2 (28.6) | 3(30) | 1.000 |
| **Previous antithrombotic medication** |  |  |  |
| **None** | 3 (42.9) | 8 (80) | 0.369 |
| **Antiplatelet** | 1 (14.2) | 1 (10) |  |
| **Anticoagulant** | 3 (42.9) | 1 (10) |  |
| **Index ischemic event** |  |  |  |
| **Baseline NIHSS score (median [IQR])** | 24 (20 – 26) | 12 (10-21) | **0.013** |
| **Prestroke mRS > 2** | 1 (14.3) | 4 (40) | **0.038** |
| **ASPECTs (median [IQR])** | 9 (7 – 10) | 9 (6-10)^*^ | 0.575 |
| **Occlusion site** |  |  |  |
| Proximal anterior circulation^§^ | 5 (71.4) | 8 (80) | 0.147 |
| Tandem occlusion | 2 (28.6) | 0 (0) |  |
| Posterior circulation | 0 (0.0) | 2 (20) |  |
| **Collateral status^$^** |  |  |  |
| Absent | 2 (28.6) | 0 (0) | 0.110 |
| Mild | 0 (0.0) | 1 (10) |  |
| Intermediate | 4 (57.1) | 3 (30) |  |
| Good | 0 (0.0) | 4 (40) |  |
| Not applicable | 1 (14.3) | 2 (20) |  |
| **TOAST classification** |  |  |  |
| ESUS | 2 (28.6) | 2 (20) | 0.521 |
| LAA | 2 (28.6) | 1 (10) |  |
| CE | 3 (42.8) | 7 (70) |  |
| **Laboratory findings** |  |  |  |
| **Leukocytes** (×10^3^/μL) (Mean ± SD) | 8.9 ± 4.4 | 12.0±3.8 | 0.172 |
| **Neutrophils** (×10^3^/μL) (Mean ± SD) | 8.1 ± 4.0 | 9.7±3.7 | 0.435 |
| **Eosinophils** (×10^3^/μL) (Median [IQR]) | 0.0 [0.0-0.0] | 0.0[0.0-0.1] | 0.156 |
| **Lymphocytes** (×10^3^/μL) (Median [IQR]) | 0.9 [0.6-1.7] | 1.4[0.9-1.8] | 0.239 |
| **NLR** (ratio) (Median [IQR]) | 8.5 [2.3-23.5] | 6.4 [4.2-10.9] | 0.828 |
| **Monocytes** (×10^3^/μL) (Median [IQR]) | 0.8 [0.7-1.0] | 0.7[0.4-1.1] | 0.569 |
| **Platelet** **count** (×10^3^/μL) (Median [IQR]) | 229 [199-346] | 224[189-371] | 0.828 |
| **INR** (Median [IQR]) | 1.1 [1.0-1.2] | 1.1 [1.1-1.3] | 0.524 |
| **D-Dimer** (µg/mL) (Median [IQR]) | 1.2 [0.9-18.4] | 3.1[1.3-5.4] | 0.345 |
| **Fibrinogen** (µg/mL) (Mean ± SD) | 272 ± 285 | 516 ± 204 | 0.072 |
| **CRP** (mg/L) (Median [IQR]) | 16.5 [1.2-56.1] | 64.0[15.6-86.4] | 0.118 |
| **Acute treatment** |  |  |  |
| **Intravenous Alteplase** | 2 (28.6) | 3 (30) | 1.000 |
| **Onset to groin time (median [IQR])** | 330 (255 – 495) | 219 (150-288) | 0.080 |
| **Number of passages** |  |  |  |
| 1 | 0 (0.0) | 7 (70) | **0.016** |
| 2-4 | 3 (42.9) | 2 (20) |  |
| ≥ 5 | 4 (57.1) | 1 (10) |  |
| **Devices** |  |  |  |
| Aspiration | 2 (28.6) | 5(50) | **0.024** |
| Stent retriever | 2 (28.6) | 0 (0) |  |
| Combined technique | 3 (42.8) | 5 (50) |  |
| **mTICI ≥ 2b** | 5 (71.4) | 10 (100) | 0.154 |
| **Outcome** |  |  |  |
| **90-day mRS > 2** | 6 (85.7) | 8 (80) | 1.000 |
|  |  |  |  |

Abbreviations. SD, standard deviation; AIS, acute ischemic stroke; ESUS, embolic stroke of undetermined source; LAA, large‐artery atherosclerosis; CE, cardioembolism; *Abbreviations. SD, standard deviation; NLR, neutrophil to lymphocyte ratio; INR, international normalized ratio; CRP, C-reactive protein.*

^§^Proximal anterior circulation includes: intracranial ICA and M1 segment of middle cerebral artery;

^$^Collaterals were assessed with Tan collateral score; *Two patients with posterior circulation ASPECT score
